# Parental control over feeding in infancy. Influence of infant weight, appetite and feeding method^☆^

**DOI:** 10.1016/j.appet.2015.04.004

**Published:** 2015-08-01

**Authors:** Alison Fildes, Cornelia H.M. van Jaarsveld, Clare Llewellyn, Jane Wardle, Abigail Fisher

**Affiliations:** a Department of Epidemiology and Public Health, Health Behaviour Research Centre, University College London, Gower Street, London WC1E 6BT, UK; b Department of Primary Care and Public Health Sciences, King's College London, Capital House, 42 Weston Street, London SE1 3QD, UK

**Keywords:** BMI, body mass index, weight SDS, weight standard deviation scores, Parental control, Pressure, Restriction, Infants, Weight, Feeding

## Abstract

*Background and objective:* Parental control over feeding has been linked to child overweight. Parental control behaviours have been assumed to be exogenous to the child, but emerging evidence suggests they are also child-responsive. This study tests the hypothesis that parental control in early infancy is responsive to infant appetite and weight. *Subjects and methods:* Participants were 1920 mothers from the Gemini twin cohort, using one randomly selected child per family. Data come from questionnaires completed when the children were approximately 8 months. Mothers completed measures of ‘pressure’ and ‘restriction’, reported feeding method (breast- and bottle feeding), rated their infant's appetite during the first 3 months, provided health professional recorded weight measurements, and reported their concerns about their infant's weight. Logistic regression examined predictors of ‘pressure’ and ‘restriction’, adjusting for maternal demographics and BMI. Interactions between feeding method and control were also tested. *Results:* ‘Pressure’ was associated with lower birth weight (OR = 0.79, 95% CI: 0.65–0.97), greater concern about underweight (OR = 1.88, 1.29–2.75), and lower infant appetite (OR = 0.59, 0.47–0.75). ‘Restriction’ was associated with higher appetite (OR = 1.44, 1.09–1.89) and bottle feeding (OR = 2.86, 2.18–3.75). A significant interaction with feeding method indicated that infants with high appetites were more likely to be restricted only if they were bottle-fed (OR = 1.52, 1.13–2.04). *Conclusion:* Mothers vary in their levels of control over milk-feeding and this is partly responsive to the infant's characteristics. They tend to pressure infants who are lighter and have a smaller appetite, and restrict infants with larger appetites if they are bottle-fed. Guidance on infant feeding may be better received if it acknowledges that parents respond to infant characteristics in order to achieve their feeding goals.

## Highlights

• Mothers vary in the control they exert over milk feeding in infancy.
• Control is associated with both feeding method and infant characteristics.
• Mothers are more likely to pressure infants who are lighter or have smaller appetites.
• Mothers are more likely to restrict bottle-fed infants who have a larger appetite.

## Introduction

Research into obesity prevention is increasingly focusing on the earliest stages of life. Rapid growth in infancy is associated with overweight in childhood, and childhood overweight is a predictor of adult obesity (Baird et al., 2005; Monteiro & Victora, 2005; Ong, Preece, Emmett, Ahmed, & Dunger, 2002). It is therefore important to understand the modifiable factors associated with early childhood weight gain. Concern has been expressed that parents are feeding in ways that increase their child's risk of weight gain, even in infancy (Farrow & Blissett, 2006; Gross, Mendelsohn, Fierman, & Messito, 2011).

Pressuring a child to eat and restricting food are the two feeding behaviours that have attracted most attention. They have been hypothesized to raise the risk of weight gain by undermining the child's ability to self-regulate food intake (Birch, Fisher, & Davison, 2003; Costanzo & Woody, 1985; Johnson & Birch, 1994). However, there is also evidence for an effect in the other direction, with appetitive or anthropometric characteristics of the child (or parental perceptions of these characteristics), influencing parental behaviour (Farrow, Galloway, & Fraser, 2009; Jansen et al., 2014; Webber, Hill, Cooke, Carnell, & Wardle, 2010). Parents who perceive their child as underweight may pressure more, while parents of overweight children may restrict their child's food intake. Recent prospective research found evidence consistent with an influence of the child's weight on parental feeding behaviours. Among 7 to 11 year old children parents were found to exert more pressure when they perceived their child to be underweight and more restriction if they perceive the child to be overweight (Webber, Cooke, Hill, & Wardle, 2010). Parents of preschool children have also been found to adapt their controlling feeding practices in response to their child's weight (Jansen et al., 2014). Similar results have been shown for appetitive behaviour, with parents exerting more pressure on a child who shows less interest in food, and being more restrictive with a child who is very food responsive (Carnell, Benson, Driggin, & Kolbe, 2014; Gross et al., 2011; Webber, Cooke, & Wardle, 2010).

Most research to date has been in pre-school (Carnell et al., 2014; Jansen et al., 2014; Johnson & Birch, 1994; Rodgers et al., 2013) or school-aged (Birch et al., 2003; Webber, Cooke, Hill et al., 2010; Webber, Hill et al., 2010) children, and few studies have investigated correlates of control behaviours in parents of young infants while they are still exclusively milk-fed. Even in infancy, the extent to which parents exert control over feeding may be related to characteristics of the child, including weight and appetite (which is measurable from early infancy) (Llewellyn, van Jaarsveld, Johnson, Carnell, & Wardle, 2011). At this stage, mothers may pressure or restrict by manipulating the frequency or quantity of milk feeds. There has been particular interest in the idea that bottle feeding gives greater scope for parental control because it is easier for the parent to monitor consumption, and parental pressure could partly explain the observed association between formula-feeding and more rapid weight gain (Mihrshahi, Battistutta, Magarey, & Daniels, 2011; Sievers, Oldigs, Santer, & Schaub, 2002; Wright, Fawcett, & Crow, 1980). However, while bottle feeding has been associated with higher feeding volumes (Hofvander, Hagman, Hillervik, & Sjolin, 1982) potentially due to coaxing the baby to ‘empty the bottle’, it has also been associated with fewer feeding occasions (Sievers et al., 2002).

The aims of this study were: (1) to investigate whether differences in maternal use of restriction and pressure at this early stage in the infant's life are associated with infant appetite and weight, and (2) to test whether mothers respond differently according to whether they are breast- or bottle-feeding. Based on studies in pre-schoolers, we hypothesized that mothers whose infant was lighter and had a smaller appetite, or who were worried about their infant being underweight, would exert more pressure; and mothers whose infant was heavier and had a larger appetite, or who were more concerned about overweight, would be more likely to restrict. We also tested the hypothesis that pressure and restriction would be higher, and less child-responsive, for bottle-fed infants.

## Methods

### Sample

Participants were mothers of twins taking part in Gemini (van Jaarsveld, Johnson, Llewellyn, & Wardle, 2010), a study of genetic and environmental influences on early growth. The Office for National Statistics (ONS) contacted all families with twins born between March and December 2007 in England and Wales (N = 6754). Families (n = 3435) who agreed to be contacted by the Gemini research team were sent a baseline questionnaire in early 2008, of whom 2402 (70%) responded and constitute the Gemini cohort. This study uses data exclusively from this baseline questionnaire. The geographic distribution of the cohort is representative of the distribution of the UK population, and the sample reflects national twin statistics on sex, zygosity, gestational age at birth, and birth weight (van Jaarsveld et al., 2010).

Participants in the present study were 1920 families with complete data on all variables included in the analysis (80% of the Gemini cohort; characteristics described below). One twin from each family was selected at random for inclusion in the analyses to avoid clustering effects. The analysis sample was comparable to the full cohort on all characteristics.

### Measures

#### Parental control

Control questions were drawn from existing parental feeding questionnaires and a review of the literature on milk-feeding on the basis that they were appropriate for mothers who were feeding from breast or bottle, and with either breast milk or formula. ‘Pressure’ and ‘restriction’ were core constructs that emerged from the review. The four ‘pressure’ items used were adapted from items in the Infant Feeding Questionnaire (Baughcum et al., 2001) and the Child Feeding Questionnaire (Birch et al., 2001). The two ‘restriction’ items were adapted from a single item by Taveras et al. (2004) (see Table 1). All items used the same 5-point response scales: never, rarely, sometimes, often, and always; with higher scores indicating higher pressure or restriction. In-depth cognitive interviews were conducted with a sample of 10 mothers with 2–6 month old infants to establish the suitability of the items and response options.

A Principal Components Analysis of the 6 items showed two factors, one with all items loading >0.6 (‘pressure’) and the other with both items loading >0.9 (‘restriction’). Internal reliability was good for the ‘restriction’ scale (alpha = 0.77), and moderate for the ‘pressure’ scale (alpha = 0.56). ‘Restriction’ and ‘pressure’ were not significantly correlated (*r* = 0.04, *p* = 0.07), indicating they measure independent constructs.

Mothers completed the control questions as part of the Gemini baseline questionnaire when the infant was around 8 months old and were asked to think back to when the infant was 3 months old and exclusively milk-fed. Less than 3% (n = 36) of all Gemini families reported introducing solids prior to 3 months. ‘Pressure’ and ‘restriction’ scores were calculated by summing the individual item scores and dividing the total by the number of completed items in the scale. For ease of interpretation, scores were dichotomized into high vs. low ‘pressure’, and high vs. low ‘restriction’, as follows: ‘High restrictors’ scored ‘often’ or ‘always’ on both ‘restriction’ items, with the rest categorized as ‘Low restrictors’. ‘High pressurers’ scored ‘often’ or ‘always’ on at least 2 of 4 items, with the rest categorized as ‘Low pressurers’. For inclusion in the analyses, missing data were allowed on no more than two ‘pressure’ items for individuals who were classified as ‘high pressurers’ on the remaining 2 items, and one ‘pressure’ item when they were ‘low pressurers’ on 3 out 4 items. On this basis, 2% of participants (n = 52) were excluded.

#### Feeding method

Feeding method was assessed with the question ‘Which feeding methods did you use in the first three months’. Response options were: ‘entirely breastfeeding’; ‘mostly breastfeeding with some bottle-feeding’; ‘equally breastfeeding and bottle-feeding’; ‘mostly bottle-feeding and some breastfeeding’; ‘almost entirely bottle-feeding (only tried breastfeeding a few times)’; ‘entirely bottle-feeding (never tried breastfeeding)’; and ‘other’. It was explained that ‘breast-feeding’ referred to feeding an infant with breast milk, either directly from the breast or expressed milk from a bottle, while bottle-feeding referred to formula milk given from a bottle. A second question was used to identify use of expressed breast milk from a bottle (‘Mostly fed directly from the breast’; ‘Equally fed from the breast and given expressed breast milk’; and ‘Mostly given expressed breast milk’).

#### Infant weight

Mothers were asked to use their child's health records (completed by health professionals but held by the mother) for birth weight and any subsequent measurements. These were used to calculate weight standard deviation scores (weight SDS) for birth and 3 months, using 1990 UK reference data (Cole, 2009; Freeman et al., 1995).

#### Weight concern

Concern about underweight was assessed using the question; ‘How concerned are you that your baby is underweight at the moment’ with response options: ‘not concerned’, ‘somewhat concerned’ and ‘very concerned’. The same phrasing and response options were used to assess current maternal concern about infant overweight. These questions were adapted from a single item from the Infant Feeding Questionnaire (Baughcum et al., 2001) and piloted with mothers of twins.

#### Appetite

Infant appetite was assessed with: ‘How would you rate your baby's appetite in the first three months’, with response options of: ‘poor’, ‘OK’, ‘good’, ‘very good’ and ‘excellent’. This item captures general appetite and has been validated against the Baby Eating Behaviour Questionnaire, a comprehensive measure of appetite during the period of exclusive milk-feeding (Llewellyn et al., 2011).

To facilitate interpretation of results, all predictor variables were dichotomized. Infant age at questionnaire completion, weight SDS at birth, and weight SDS at 3 months were normally distributed and divided at the sample mean. Feeding method was divided into ‘breastfed’ (‘entirely breastfeeding’ or ‘mostly breastfeeding with some bottle-feeding’) and ‘bottle-fed’ (all other categories including mixed feeding and expressed milk in a bottle). Maternal concern about underweight and overweight were grouped as ‘not concerned’ vs. ‘concerned’. ‘Concerned’ included ‘somewhat’ and ‘very’ concerned for both underweight and overweight. Appetite was dichotomized into ‘low’ (response options ‘poor’ and ‘OK’) vs. ‘high’ (response options ‘good’, ‘very good’ and ‘excellent’).

Mothers reported their date of birth and educational qualifications. Self-reported height and weight was used to calculate maternal BMI (kg/m^2^). Maternal age and BMI were treated as continuous variables, while education was grouped as ‘university level’ vs. ‘below university level’.

### Statistical analyses

Correlates of maternal control were analysed using logistic regression. Associations between maternal control, infant characteristics, and feeding method were examined using separate regression models for each infant characteristic, controlling for maternal BMI, age and education (Model 1). All the infant characteristics were then included simultaneously (Model 2). Interactions between feeding method and control were tested and regression analyses were repeated including significant interactions between feeding method and child variables as a covariate. Analyses using all the variables continuously where appropriate gave the same results, so only the analyses that included the dichotomized variables are presented for ease of interpretation. An infant's sucking reflex develops at 34 weeks and this may influence feeding behaviour so analyses were repeated excluding all infants born before 34 weeks gestation but results remained unchanged. All analyses used SPSS Version 20 for Windows.

## Results

Sample characteristics are shown in Table 2. Mothers were on average 34 years old, just under half (44%) were university educated, and their mean BMI was 25. Approximately one third (30%) of infants were ‘mostly’ or ‘entirely’ breastfed during the first 3 months. Mean infant birth weight SDS (−0.56) and weight SDS at 3 months (−0.27) were less than the 1990 UK reference value, as expected for a twin sample. A minority of mothers (7%) reported concern about the baby being underweight. Very few (2%) were concerned about overweight so this was excluded from the analyses. The majority (78%) of infants were described as having ‘good’, ‘very good’ or ‘excellent’ appetite.

The mean ‘pressure’ score was 2.92 (SD = 0.69) and mean ‘restriction’ score was 2.87 (SD = 1.28). Scores were normally distributed and covered the full range (1 to 5). Using the categorization described above, just over a third of mothers (38%) were in the high ‘pressure’ group, and just over a quarter (27%) in the high ‘restriction’ group.

### Predictors of ‘pressure’

Results of the logistic regression models predicting ‘pressure’ are shown in Table 3. In Model 1, lower infant birth-weight, lower infant appetite, and greater maternal concern about infant underweight were each associated with higher ‘pressure’. In Model 2, all the infant variables and feeding method were included simultaneously to examine their independent associations with ‘pressure'. As predicted, mothers were less likely to use pressure in feeding if their infant had a heartier appetite (OR = 0.59, 95% CI: 0.47–0.75), or a higher birth weight (OR = 0.79, 95% CI: 0.65–0.97), and were more likely to use pressure if they were concerned about the baby being underweight (OR = 1.88, 95% CI: 1.29–2.75). Interactions between feeding method and infant variables were not significant.

### Predictors of ‘restriction’

Associations between infant characteristics, feeding method and ‘restriction’ (controlling for maternal characteristics) are presented in Table 4. Model 1 shows the associations adjusted for maternal anthropometric and demographic characteristics. Higher infant appetite and lower maternal concern about underweight were related to ‘restriction’. Mothers were also more likely to restrict if they used bottle-feeding rather than breastfeeding. In the multivariate model (Model 2) infant appetite continued to relate to ‘restriction’ (OR = 1.44, 95% CI: 1.09–1.89), but the negative association with concern about underweight no longer reached significance. Bottle-feeding continued to be associated with higher ‘restriction’ (OR = 2.86, 95% CI: 2.18–3.75).

Because the interaction between feeding method and infant appetite approached significance (β = 0.548 (SE = 0.324), *p* = 0.091), the multiple logistic regression analysis for ‘restriction’ was repeated stratifying by feeding method. This showed that mothers who bottle-fed their infants restricted more in response to a heartier infant appetite (OR = 1.52, 95% CI: 1.13–2.04, *p* = 0.006), but there was no significant association between appetite and ‘restriction’ in breastfeeding mothers.

All analyses were repeated selecting the other twin from each pair and the results remained consistent.

## Discussion

Consistent with our hypotheses, the results of this study showed that mothers whose infant had a lower birth weight or lower appetite, or were more concerned about the infant being underweight were more likely to use pressure in feeding. Mothers whose infant had a higher appetite were more likely to restrict, but there was no association with weight. These results suggest that characteristics of the infant in early life influence the mother's way of feeding.

We found no evidence that bottle-feeding mothers exerted more pressure, but they were more likely to be restrictive if they had an infant with a good appetite. Perhaps the messages against over-feeding with the bottle have permeated and this has now extended to parents being more discriminating (and therefore apparently restrictive) when they offer bottles. Bottle-fed infants are fed less often than breast-fed infants, and with longer intervals between feeds (Sievers et al., 2002). A recent study found that feeding on a schedule was associated with more rapid weight gain (Mihrshahi et al., 2011), which the authors suggested could be due to overriding the infant's ability to self-regulate. However our finding that bottle-feeding mothers are restrictive if they have an infant with a good appetite suggests that bottle feeding mothers remain responsive to the infant.

The finding that breastfeeding mothers are less likely to restrict is supported by previous research suggesting that longer duration of breastfeeding is associated with less restrictive feeding behaviour at 1 year of age (Taveras et al., 2004). Longitudinal research is needed to investigate whether restriction during the period of exclusive milk feeding persists following introduction of solids, and to test whether restriction in early infancy relates to later weight, independent of appetite and early weight trajectory.

The finding that mothers exert more pressure if the baby has a smaller appetite is similar to results from a recent study of infants up to 6 months old, although in that study, not all infants were exclusively milk-fed (Gross et al., 2011). No direct association between feeding method and pressure was observed in the present study, and feeding method did not moderate the relationship between any of the infant characteristics and pressure. Interestingly, an inverse relationship between maternal encouragement of bottle emptying and infant risk for excess weight gain has been observed previously (Li, Fein, & Grummer-Strawn, 2008). While we cannot exclude the possibility that bottle-feeding may result in an overriding of appetite self-regulation in some infants, our results suggest that any associations with weight are likely due to mechanisms other than maternal pressure. Alternative feeding mechanisms contributing to this association could include sucking pressure and milk flow (Mizuno & Ueda, 2006).

When asked directly, fewer than 2% of mothers expressed any concern about overweight in their infants. Because the infants in the present study were twins (albeit one randomly selected from each twin pair), they tended to have lower weights at birth and 3 months, and therefore mothers may be less concerned about overweight than the general population. However, there is currently no clinical definition of overweight before age two years in the UK; rapid weight gain is used to identify infants at risk of obesity (Dennison, Edmunds, Stratton, & Pruzek, 2006; Simmons, 2008). In the absence of criteria for assigning overweight status at any one time point, it is not surprising that parents do not express concern. Numerous studies have shown that parents fail to recognize overweight or obesity in their children (Carnell, Edwards, Croker, Boniface, & Wardle, 2005; Laraway, Birch, Shaffer, & Paul, 2010; Towns & D'Auria, 2009), and a recent study found that mothers of overweight toddlers were more likely to be satisfied with their child's weight (82%) than mothers of healthy weight (72%) or underweight toddlers (32%) (Hager et al., 2012). Our results revealed greater maternal concern about underweight than overweight, although this may have more reality in a twin sample. It is worth noting however that only 7% of mothers reported concern about infant underweight, suggesting that the sensitivity of this predictor in the models may have been quite low. Arguably this makes the significant association between concern about underweight and pressure in feeding observed in the present study more interesting.

### Strengths and limitations

This study had a number of strengths. The sample was large with a wide range of appetite and weight in the infants. The majority (96%) of the infant weight data were from the child health records measured and recorded by health professionals and rates of exclusive breast and bottle-feeding were consistent with the UK population (Bolling, Grant, Hamlyn, & Thornton, 2007). However, the participants were families with twins and therefore infant weights at birth and 3 months are low in comparison with the general population; although this should not substantially affect associations with maternal behaviours. Additionally it is unclear whether fathers or additional carers play a larger role in the early feeding of twins compared to singleton infants. Additional maternal characteristics such as mothers' own appetitive behaviours or disordered eating have been associated with the use of control feeding in older children (Blissett & Haycraft, 2011; McPhie, Skouteris, Daniels, & Jansen, 2014), and may have influenced the relationship between control feeding and infant appetite in this study. Future research should consider including additional measures of parental characteristics when exploring parental control in infant feeding.

Appetite scores were parent-reported and retrospective, so could be subject to bias, and they were collected at the same time as information on maternal control. However, birth weight pre-dated the maternal control data; supporting a tentative causal conclusion. The internal consistency of the ‘pressure’ scale was below the accepted level of 0.7, and the ‘restriction’ scale only had two items; both these features would reduce reliability, but it is likely this would underestimate the true effects. Future research would benefit from the development of improved measures of control feeding in early infancy.

### Future research and implications

Longitudinal studies of feeding behaviour are important to fully understand the direction of the relationship between maternal feeding behaviour with appetite and weight. Investigating differences in feeding behaviour between siblings could shed further light on child-specific characteristics that predict feeding strategies (Farrow et al., 2009; Webber, Cooke, & Wardle, 2010).

Evidence that even during the phase of milk-feeding mothers appear responsive to their child's appetite and weight in their use of pressure or restriction has implications for health professional advice. It could be helpful for health professionals to acknowledge that mothers are responding to characteristics of their infant in the ways that they feed. Advice could therefore be adapted where appropriate and may be received better and implemented more effectively.

### Conclusion

This is one of the first studies to investigate the infant predictors of parental control in the feeding domain in early infancy. The findings indicate that maternal pressure or restriction is associated with both feeding method and the characteristics of the infant. The results are consistent with findings in older children which suggest that parents adapt their feeding strategies to the characteristics of the child to achieve their feeding goals.

## Figures and Tables

**Table 1:** ‘Pressure’ and ‘restriction’ questionnaire items.

Scale items^a^
‘Pressure’
If my baby stopped feeding, I let him/her have a break then tried again a bit later
If my baby stopped feeding, I tried other methods to encourage him/her e.g. moved baby into a different position or switched breasts
If my baby did not feed much on one occasion, I offered him/her another feed a bit sooner than I normally would
If my baby did not feed much on one occasion, I made sure he/she took a larger amount at the next feed
‘Restriction’
I was careful not to feed my baby too frequently
I was careful not to feed my baby too large an amount

a All items measured using a 5 point response scale: ‘never’, ‘rarely’, ‘sometimes’, ‘often’, ‘always’; with higher scores indicating higher ‘pressure’ or ‘restriction’.

**Table 2:** Sample characteristics.

	N	%	Mean	(SD)
Infant-related variables	1920			
Age in months^a^			8.10	(2.18)
Younger (<8.11 months)	1081	56.3		
Older (≥8.11 months)	839	43.7		
Sex				
Male	950	49.5		
Female	970	50.5		
Birth weight SDS			−0.56	(0.93)
Lower (≤−0.56)	968	50.4		
Higher (>−0.56)	952	49.6		
3 month weight SDS			−0.27	(1.06)
Lower (≤−0.26)	960	50.0		
Higher (>−0.26)	960	50.0		
Concern about infant's underweight			1.04	(0.27)
No concern	1785	93.0		
Concern	135	7.0		
Concern about infant's overweight			1.02	(0.14)
No concern	1884	98.1		
Concern	36	1.9		
General appetite rating			3.50	(1.15)
Lower (poor, ok)	416	21.7		
Higher (good, very good, excellent)	1504	78.3		
Feeding method during first three months				
Breastfed	574	29.9		
Bottle fed	1346	70.1		
Gestational age (weeks)			36.25	(2.41)
At term (≥37 weeks)	834	43.4		
Pre-term (<37 weeks)	1086	56.6		
Maternal variables				
Age in years			33.75	(5.05)
Educational level				
Below university	1083	56.4		
University	837	43.6		
BMI in kg/m^2^			25.19	(4.88)
Number of older children				
0	996	53.5		
1 or more	864	46.5		
Parental feeding behaviours				
‘Pressure’			2.92	(0.69)
Lower	1189	61.9		
Higher	731	38.1		
‘Restriction’			2.87	(1.28)
Lower	1407	73.3		
Higher	513	26.7		

a Age in months at questionnaire completion.

**Table 3:** Associations of infant related variables with increased ‘pressure’.

	Model 1^a^			Model 2^b^		
	OR	95% CI	p value	OR	95% CI	p value
Age						
Younger	1			1		
Older	1.05	0.87–1.27	0.591	1.03	0.85–1.24	0.800
Sex						
Male	1			1		
Female	1.09	0.90–1.31	0.384	1.06	0.88–1.28	0.551
Birth weight SDS						
Lower	1			1		
Higher	**0.75**	**0.63–0.91**	**0.003**	**0.79**	**0.65–0.97**	**0.023**
3 month weight SDS						
Lower	1			1		
Higher	0.85	0.70–1.02	0.085	1.08	0.87–1.33	0.488
Appetite rating						
Lower	1			1		
Higher	**0.53**	**0.43–0.67**	**<0.001**	**0.59**	**0.47–0.75**	**<0.001**
Concern about underweight						
No concern	1			1		
Concern	**2.43**	**1.70–3.47**	**<0.001**	**1.88**	**1.29–2.75**	**0.001**
Feeding method						
Breastfed	1			1		
Bottle-fed	1.22	0.99–1.51	0.062	1.14	0.91–1.41	0.251

a Model 1 = Separate logistic regression models for each infant characteristic, controlling for maternal age, education, BMI and feeding method.

b Model 2 = A single logistic regression model including all infant variables simultaneously, controlling for maternal age, education, BMI and feeding method. Significant values (at an alpha level of p<0.05) are **bolded**.

**Table 4:** Associations of infant related variables with increased ‘restriction’.

	Model 1^a^			Model 2^b^		
	OR	95% CI	p value	OR	95% CI	p value
Age						
Younger	1			1		
Older	1.05	0.85–1.29	0.679	1.06	0.86–1.31	0.594
Sex						
Male	1			1		
Female	0.92	0.75–1.14	0.449	0.93	0.76–1.15	0.492
Birth weight SDS						
Lower	1			1		
Higher	0.93	0.76–1.15	0.520	0.89	0.71–1.12	0.313
3 month weight SDS						
Lower	1			1		
Higher	1.05	0.85–1.29	0.671	0.99	0.78–1.24	0.895
Concern about underweight						
No concern	1			1		
Concern	**0.55**	**0.34–0.87**	**0.011**	0.62	0.38–1.00	0.052
Appetite rating						
Lower	1			1		
Higher	**1.53**	**1.18–2.00**	**0.002**	**1.44**	**1.09**–**1.89**	**0.010**^c^
Feeding method						
Breastfed	**1**			**1**		
Bottle-fed	**2.71**	**2.08–3.54**	**<0.001**	**2.86**	**2.18–3.75**	**<0.001**^c^

a Model 1 = Separate logistic regression models for each infant characteristics, controlling for maternal age, education, BMI and feeding method.

b Model 2 = A single logistic regression model including all infant variables simultaneously, controlling for maternal age, education, BMI and feeding method.

c Interaction between feeding method and infant appetite (β = 0.548, (SE = 0.324), *p* = 0.091). Stratified analyses by feeding method showed a significant association between appetite and ‘restriction’ in bottle-feeding mothers (OR = 1.52, 95% CI: 1.13–2.04, *p* = 0.006), but not in breastfeeding mothers (OR = 1.08, 95% CI: 0.53–2.20, *p* = 0.840). Significant values (at an alpha level of p<0.05) are **bolded**.
